# P-672. Comparative Risk of Major Adverse Cardiovascular Events Following RSV versus Influenza Hospitalization in the United States: A MarketScan Database Retrospective Cohort Study

**DOI:** 10.1093/ofid/ofaf695.885

**Published:** 2026-01-11

**Authors:** Julia Yang, Steven M Smith, Tianze Jiao, Kenneth Smith, Debbie-Ann Shirley, Venugopalan Veena, Nicole M Iovine, Nirma Khatri Vadlamudi

**Affiliations:** University of Florida, Gainesville, Florida; University of Florida, Gainesville, Florida; University of Florida, Gainesville, Florida; University of Pittsburgh, Pittsburgh, Pennsylvania; University of Florida, Gainesville, Florida; University of Florida, College of Pharmacy, Gainesville, Florida; University of Florida, Gainesville, Florida; University of Florida, Gainesville, Florida

## Abstract

**Background:**

Respiratory syncytial virus (RSV) and influenza are both common causes of acute respiratory illness, particularly in older adults and individuals with underlying health conditions. While the association between influenza and major adverse cardiovascular events (MACE) has been well documented, the cardiovascular impact of RSV remains less well studied. This study aims to compare the cardiovascular risks associated with RSV versus influenza among U.S adults.

**Methods:**

We conducted a retrospective cohort study using the 2018-2022 Merative MarketScan® Commercial Inpatient Claims Database to patients aged 18 to 64 years hospitalized with a diagnosis of either RSV or influenza. The index date was the first inpatient admission for RSV or influenza during the study year. The primary outcome was the incidence of MACE, a composite outcome of myocardial infarction, stroke, or heart failure, within 30 days, 90 days, and 1-year following the index RSV or influenza-associated hospitalization. Logistic regression was used to estimate odds ratios (ORs) and 95% confidence intervals (CIs) for the association between viral infection and the risk of MACE, adjusted by sex and age group (18–49 years and ≥50 years).

**Results:**

We identified adults hospitalized with RSV (n = 1,898) or influenza (n = 7,972) between 2018 and 2022. Compared to influenza, RSV infection was associated with a significantly higher risk of MACE across all follow-up periods (1-year adjusted (a) OR: 1.35; 95% CI: 1.17–1.55). Among individual components, heart failure (HF) showed a significantly elevated risk across any time point (1 year aOR: 1.41; 95% CI: 1.21–1.64), while no significant differences were observed for AMI or stroke.

**Conclusion:**

Moderate-to-severe RSV infections were associated with a higher risk of MACE compared to influenza, particularly driven by an increased risk of heart failure within one year. These findings highlight the importance of post-RSV cardiovascular monitoring and may inform public health strategies.

**Disclosures:**

Venugopalan Veena, PharmD, Merck: Grant/Research Support Nirma Khatri Vadlamudi, PhD, MPH, Sanofi: Advisor/Consultant|Sanofi: Honoraria

